# Development and validation of a questionnaire to evaluate lifestyle-related behaviors in elementary school children

**DOI:** 10.1186/s12889-015-2248-6

**Published:** 2015-09-16

**Authors:** G. Santos-Beneit, M. Sotos-Prieto, P. Bodega, C. Rodríguez, X. Orrit, N. Pérez-Escoda, R. Bisquerra, V. Fuster, JL Peñalvo

**Affiliations:** Foundation for Science Health and Education, Avda. Diagonal 442, 3°, 1ª, 08037 Barcelona, Spain; Department of Nutrition, Harvard T.H. Chan School of Public Health, Boston, MA USA; GROP (Grup de Recerca en Orientació Psicopedagògica), Departament MIDE (Mètodes d’Investigació i Diagnòstic en Educació), Universitat de Barcelona, Barcelona, Spain; Fundación Centro Nacional de Investigaciones Cardiovasculares (CNIC), Madrid, Spain; Icahn School of Medicine at Mount Sinai, New York, NY USA; Friedman School of Nutrition Science and Policy at Tufts University, Boston, MA USA

## Abstract

**Background:**

The SI! Program promotes cardiovascular health through a multilevel school-based intervention on four lifestyle-related components: diet, physical activity, understanding the body and heart, and management of emotions. We report here the development and validation of the KAH (knowledge, attitudes and habits)-questionnaire adapted for elementary school children (6–7 years old) as a tool for the forthcoming evaluation of the SI! Program, where the KAH scoring will be the primary outcome. The efficacy of such an intervention will be based on the improvements in children’s KAH towards a healthy lifestyle.

**Methods:**

The questionnaire validation process started with a pool of items proposed by the pedagogical team who developed the SI! Program for elementary school. The questionnaire was finalized by decreasing the number of items from 155 to 48 using expert panels and statistical tests on the responses from 384 children (ages 6–7). A team of specialized psychologists administered the questionnaire at schools providing standard directions for the final administration. The internal consistency was assessed using Cronbach’s α coefficients. Reliability was measured through the split-half method, and problematic items were detected applying the item response theory. Analysis of variance and Tukey’s test of additivity were used for multiple comparisons.

**Results:**

The final KAH-questionnaire for elementary school children should be administered to children individually by trained staff. The 48 items-questionnaire is divided evenly between the 4 components of the intervention, with an overall Cronbach’s α = 0.791 (α = 0.526 for diet, α = 0.537 for physical activity, α = 0.523 for human body and heart, and α = 0.537 for management of emotions).

**Conclusions:**

The KAH-questionnaire is a reliable instrument to assess the efficacy of the SI! Program on instilling healthy lifestyle-related behaviors in elementary school children.

**Electronic supplementary material:**

The online version of this article (doi:10.1186/s12889-015-2248-6) contains supplementary material, which is available to authorized users.

## Background

Cardiovascular diseases are the leading cause of death worldwide [1]. It is known that obesity and its related conditions such as diabetes and hypertension are major contributors to the development of cardiovascular disease, and these conditions result from inadequate physical activity and dietary habits [2]. The need for a comprehensive approach in addressing this issue is widely recognized [3, 4]. The SI! Program is a school-based intervention to promote cardiovascular health from early childhood [5–7]. Through a comprehensive view of health promotion, the Program tackles four inter-related components: diet (D), physical activity (PA), human body and heart (HB), and emotions management (E) [5]. As a long-term intervention, the SI! Program was designed to cover the entire compulsory education in Spain (3–16 years old) from kindergarten to secondary education. The Program is developed in successive steps that tailor the intervention to the developmental level of children. Strategies and materials are also adapted to the compulsory curriculum of the school. Children are the main focus of the SI! Program. Besides classrooms activities, the Program is reinforced by involving teachers, families and the school environment to reach the children’s immediate circles. Teachers receive formal training, a teaching guide, and have access to all the materials and resources available on the SI! Program web site. During the intervention, families receive instructions for home activities and key messages about their children’s health. The school environment is also involved mainly through an annual Healthy Fair organized by the school staff. In addition, the SHE Foundation provides a series of recommendations for a healthy school setting.

The core intervention of the SI! Program for preschoolers (1st to 3rd level, age 3–5) is based on three teaching units per year focused on D, PA and HB, respectively, whereas the E component is implemented across the curriculum. All the materials were specifically developed by the Department of Pedagogy at the SHE Foundation. The SI! Program for preschool follows a meaningful learning approach: games, experiential activities, stories, audiovisual resources, etc.

The SI! Program for elementary (1st to 6th level, age 6–11) also adapts the strategies and materials to the compulsory curriculum at that educational level. The core intervention includes classroom activities grouped in what we called “healthy challenges” related to D, PA, HB, and E. These challenges are distributed across the different levels and implemented by the corresponding teachers through different subjects (science, physical education, music, art classes…). The Autonomous University of Barcelona’s Institute of Education Sciences has collaborated in the development of these materials.

The efficacy of the first phase of the SI! Program (preschool, ages 3–5) was evaluated through a cluster-randomized controlled trial [5] in which an adapted version of the questionnaire previously used in the Colombian Initiative for Healthy Heart Study for children aged 3 to 5 was utilized [8]. The questionnaire assesses the knowledge, attitudes and habits (KAH) of children towards a healthy lifestyle mostly following the theory of the Transtheoretical Model of Change [9] and assuming a progressive acquisition and retention of healthy habits in children [5, 8]. The KAH system has been applied in previous school-based interventions on health promotion [10–14]. The system has proven to serve as intermediate indicator of improved lifestyle and, therefore, as a success measure for the ability of the intervention to instill these concepts and provide children with tools for self-promotion of health.

Considering the long-term intervention of the SI! Program, assessment tools are also developed successively and adapted to the stages of maturation of children. The aim of this paper is to describe the development and validation of the KAH-questionnaire for the forthcoming evaluation of the SI! Program for elementary intervention (children aged 6 to 11), since in the previous Colombian study the questionnaire was developed for children aged 3 to 5 years.

## Methods

### Participants and study design

The development of the questionnaire and validation study took place in two stages during the academic years of 2012–13 and 2013–14 in three different locations in Spain. A total of 384 children from two schools in Catalonia and five schools in Madrid participated in the study. In Catalonia one school from the town of Manresa was recruited (never-exposed 6 year old children, *n* = 44). Likewise, one school from the town of Cardona (Catalonia) involved in the SI! Program participated in the study (ongoing intervention, 7 year old children, *n* = 45). In the city of Madrid, five similar schools were recruited (never-exposed 6 year old children, *n* = 295). The recruitment was conducted by SI! Program coordinators of the corresponding locations. The study received the approval from the Regional Ethics Committee for Clinical Research (CEIC-R) of Madrid as well as from the Regional Education Authorities of Catalonia and Madrid, including the schools’ principals. Informed consent was required from parents or legal guardians to participate in the study. Collected data were treated according to the Organic Law 15/1999 for the Protection of Personal Data, ensuring the confidentiality of all of the information submitted by the participants. We have followed the qualitative research review guidelines (RATS) for reporting the results of this study.

### Basis of the questionnaire

**Fig. 1:**
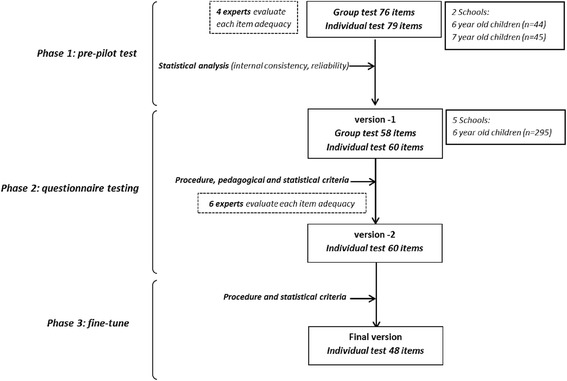
Stages of the progressive development of the KAH-questionnaire for elementary school children

The questionnaire is based on the Transtheoretical Model of Change [9]. We restructured the five phases of the model into our own by merging the “precontemplation” and “contemplation” stages into the acquisition of knowledge (K); the “preparation” phase corresponds to the attitude to change (A); finally, “action” and “maintenance” were merged into the acquisition of the habit (H). The questionnaire was specifically built to score each domain (KAH) and each component (D, PA, HB, E) providing four component specific-KAH scores (D, PA, HB, E), plus a composite score (overall KAH). Based on the materials and strategies of the SI! Program for children in elementary schools, a pool of 160 items was proposed by a multidisciplinary team of experts. Items were phrased according to specific KAH domains and were thoroughly revised for consistency with the developmental level of children. From this starting point, a progressive improvement of the questionnaire was carried out as described in the results section (Fig. 1).

### Statistical analysis

Descriptive statistics (frequency tables and histograms) were generated for each item. The internal consistency of the items (overall and by component) was assessed using Cronbach’s α coefficients. Reliability was measured through the split-half method, and problematic items were detected applying the item response theory (IRT). ANOVA and Tukey’s test of additivity were used for multiple comparisons. Data were analyzed using SPSS version 21 for Windows (SPSS Inc., Chicago IL, USA).

## Results

### Phase 1: Pre-pilot test

Four members from the SI! Program pedagogical team evaluated the initial 160 items using a Likert scale of 0–10. The agreement of each item to the contents of SI! Program was assessed for each component. The adequacy of the pictures and wording, and the agreement between the answers to the proposed questions were also evaluated. After modifications, a 155-item questionnaire was released divided into individual (79 items) and group (76 items) blocks. The questionnaire was applied simultaneously in two towns of the Catalonian region (Manresa and Cardona). To assess the ceiling and floor effect, the questionnaire was tested in a never-exposed school (Manresa) and in an intervened school (Cardona).

The individual 79-item questionnaire was administered in 25–35 min, while the 76-item group test was administered in the classroom in 80–100 min. Each item scored from 0 to 3, and each question had 4 possible responses. To reduce the number of items, several tests and reliability analyses were carried out. Frequency tables and histograms were generated for each item to analyze distractors and evaluate potential improvements in response’s options. Through ANOVA, the differences between items in both individual and group tests were confirmed (*p* < 0.001). Tukey’s test of additivity was used to confirm that participants with high scores tended to score high on all individual items (and vice versa) for the test group (*p* = 0.408), but not for the individual test (*p* < 0.05). Using IRT, the probability of success/failure on each item according to the overall score was graphically displayed, and problematic items were also detected. Reliability analyses were used to eliminate items with negative or very low values. After this initial assessment, Cronbach's α increased from 0.797 to 0.849 for the group test, and from 0.815 to 0.885 for the individual test. The resulting version-1 of the questionnaire consisted of 58 items for the group test (average 60 min administration) and 60 items for the individual test (average 30 min administration).

### Phase 2: Questionnaire testing

The version-1 of the questionnaire was administered to 6 year old children never exposed to SI! Program from five schools in the city of Madrid (*n* = 295). The group block was administered in the classrooms following a standardized protocol [14, 15] where a trained psychologist read the questions aloud and utilized slide presentation as supporting material. The application of the questionnaire was also monitored by the school teachers to help answer questions and ensure children’s attention. However, children in this age range were easily distracted and had a low reading literacy. Thus, the difficulty of effectively managing the group questionnaire prompted the decision to exclude that approach, and also relied on the good results obtained for the individual applications.

Items from individual and group questionnaires were therefore merged, and those items with low correlation were excluded. Some items were rewritten to redistribute questions on KAH across the four different components (D, PA, BH, E), resulting in the version-2 of the questionnaire. The pedagogical team of SI! Program reviewed the second version, and a panel of external experts consisting of educators, teachers, and psychologists was appointed to finally evaluate the questionnaire. Response options were reduced and questions and illustrations were simplified. The process resulted in version-2 composed of 15 items per component and 5 per domain. Later, a 60-item questionnaire (20 min duration) was administered to the same children after 7 months.

### Phase 3: Fine-tuning

**Fig. 2:**
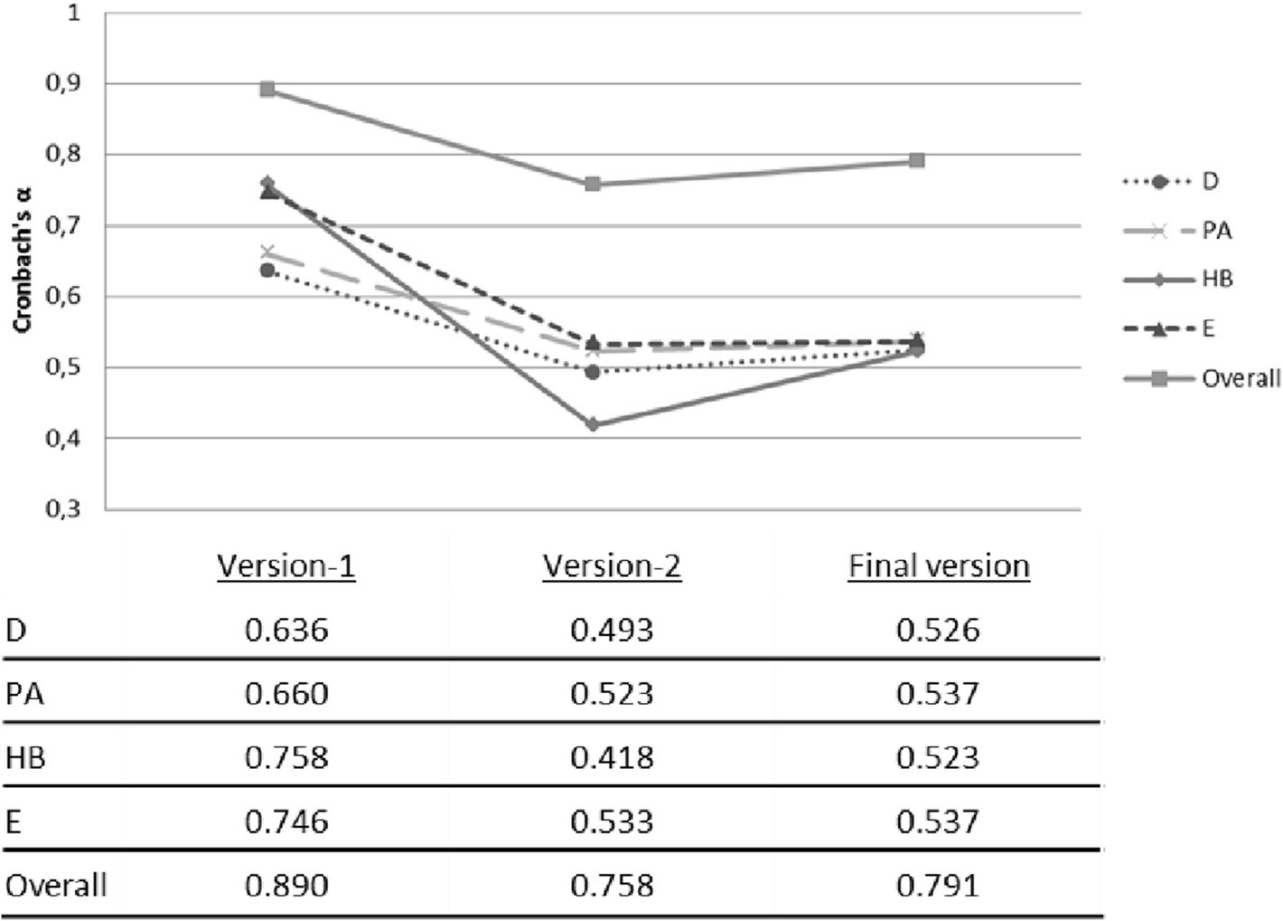
Overall and component-specific Cronbach’s α on the different versions of the KAH-questionnaire *D: diet, PA: physical activity, HB: human body and heart, E: emotions*

After administering the version-2 questionnaire, the item-corrected correlation was assessed. Those items with poor relationship between the item score and the overall score were eliminated. The approach improved Cronbach’s α (Fig. 2). The final items were reformulated to redistribute between the domains and components (4 items on each domain and each component; 48 items, 12 per component).

The final structure of the questionnaire was conceived following the indications of the psychologists who administered the questionnaires in the schools. The layout of the questionnaire consists of a cartoon character introducing each question over the story to keep the children’s attention. Correct answers add two points to the final score, one point for intermediate responses and 0 points for incorrect answers. The component specific-KAH scores ranged from 0 to 24 (D-KAH, PA-KAH, HB-KAH, E-KAH), and the overall-KAH scores from 0 to 96 points. Table 1 shows some examples of the final questionnaire, and the whole text for the questionnaire is shown in the Additional file 1.

**Table 1:** Sample questions of the final KAH-questionnaire version

COMPONENT	DOMAIN (K-A-H)	RESPONSE OPTIONS (scoring)
Diet	K: How often should you eat vegetables?	Never (0)
		Sometimes (1)
		Every day (2)
	A: What do you do if you have to try a new food?	I always try it (2)
		I try it sometimes (1)
		I never try new food (0)
	H: How often do you eat pastries?	Every day (0)
		Once a week (1)
		Only at birthdays or parties (2)
Physical activity	K: How fast should the heart beat after running?	It should beat slowly (0)
		It should beat fast (2)
		It always beats to the same rhythm (0)
	A: How do you feel when you have to exercise?	I always feel happy (2)
		Sometimes I feel lazy or tired (1)
		I never feel good (0)
	H: What do you usually do after school?	I play videogames or watch TV (0)
		I play sitting down (0)
		I play around (run, jump, climb…) (2)
Human body and heart	K: What do you release when you sweat?	Blood (0)
		What you have drunk (0)
		Water and waste (2)
	A: What do you think about adults who smoke?	I don’t care (0)
		It’s wrong and I ask them to stop (2)
		It’s wrong but I don’t say anything (1)
	H: How often do you bath or shower?	Every night (2)
		Every other day (1)
		Only on weekends (0)
Emotions	K: When do people blush?	When they are embarrassed (2)
		When they are happy (0)
		When they are sad (0)
	A: What do you usually do when your parents ask you to help out at home?	I always help (2)
		I protest, but I help (1)
		I never help (0)
	H: What do you usually do when someone is bothering a friend?	Nothing (0)
		I try to help (2)
		I fight it out (0)

*K* knowledge, *A* attitudes, *H* habits

## Discussion

Validation is needed to guarantee trustworthiness of the questionnaire to the effects of a school-based intervention and, therefore, achieve a reliable and successful evaluation. The Transtheoretical Model of Change [9] has been used to evaluate and create the KAH scores. During the questionnaire application, the K, A or H domain should be kept in mind by the psychologists to get the most accurate response, but is no revealed to the children to avoid bias by self-awareness [16].

Previous interventions on children between 6 and 7 years old have also used the KAH system. The Pathways Study developed its own KAH-questionnaire for the four components of their intervention: physical activity, diet, attitudes towards weight and cultural identity [14]. The CATCH Study also included questionnaires on food knowledge, attitudes, dietary habits, social support and self-confidence [10]. The Cronbach’s α values obtained in both studies were in line with our results. The KAH system has been also applied in older children and adults on an intervention to promote wellbeing within schools with similar results (α ranging from 0.53–0.82 for nutrition education, food and beverage knowledge, activity communication and physical assessment components) [17]. When individual components were analyzed, Cronbach’s α values show a moderate internal consistency (close to 0.53). These values were also observed in other studies analyzing individual components separately, i.e. for diet, physical activity [18] or knowledge about food [19]. A questionnaire developed for adolescents, which included similar components to the SI! Program (social support, responsibility for health, eating habits and physical activity, stress management), obtained higher α values for component (0.75-0.88) and also reached a higher value when was taken as a whole (0.9) [20].

The KAH based-questionnaire has been used in the Colombian experience of the SI! Program for preschool (Colombian Initiative for Healthy Hearts Study) [8], capturing a 10.9 % increase in the KAH-score of the intervened children versus 5.3 % in control group. Similarly, the cluster-randomized controlled trial carried out with Spanish preschoolers found significant improvements in the KAH-score of intervened children after 1-school year both in the overall KAH (3.45, 95 %CI, 1.84-5.05) and component-specific KAH scores (D: 0.93, 95 %CI, 0.12-1.75; PA: 1.93, 95 %CI, 1.17-2.69; HB: 0.65, 95 %CI, 0.07-1.24) [6]. These positive results, and the ability of the questionnaire to capture changes over time, suggest that the KAH system is a reliable instrument to evaluate the effectiveness of the SI! Program in the elementary schools. The efficacy of the SI! Program to instill a healthy lifestyle among elementary school children will be evaluated through a randomized controlled trial where follow-up measurements will be performed at 3 and 6 years from baseline. Questions regarding the K domain will be progressively adapted to the curricular level at each follow-up (9 and 11 years old children) while maintaining the same questionnaire structure.

The trained interviewers (pediatric psychologists) played a fundamental role in the study. They had to detect the social desirability responses and conduct the interview to get the best possible answer. Also, as noted above, the purpose of the questionnaire and the K, A or H domain should be kept in mind to get the most accurate response. This limitation is addressed by providing instructions on how to administer the questionnaire in a standardized manual and a training period conducted by the scientific team at the SHE Foundation. Besides, the randomized design of the forthcoming trial allows managing the possible bias.

The use of the questionnaire outside these settings might require additional validation since we have developed a highly specific questionnaire for elementary school children, adapted to the contents of the public elementary education in Spain. However, the questionnaire covers a global view of health, including key points as physical activity (and sedentary time), healthy diet (promote eating vegetables and fruits…), body and heart (hygiene) and emotions (recognition of self and external emotions); and could be a useful tool due to its simplicity. Therefore, this questionnaire can be used for screening, monitoring and evaluating health related surveys.

The process of developing specific questionnaires to assess behavior change requires attention to not only specific aspects of the intervention but also other areas related to children’s development (e.g. psychology or pedagogy). In our case, the vocabulary and the duration of the assessment were carefully considered during the development of the questionnaire. Efforts were made to reduce the number of items, and to adapt vocabulary and administration procedure to the school level.

## Conclusions

The process of developing specific questionnaires requires a multidisciplinary team with experts in the fields of the intervention and in other areas related to children’s cognitive skills (e.g. psychology or pedagogy). Characteristics of the questionnaire such as the number of items, the number of possible answers to each question, the vocabulary used, or the distribution of the questions, are very important factors to consider in children surveys. The evaluation system based on the assessment of knowledge, attitudes and habits of children is a reliable tool for measuring the efficacy of SI! Program intervention in elementary school students.

## Additional file

Additional file 1:
**Appendix.** (DOC 131 kb)

## Abbreviations

KAH: Knowledge, attitudes and habits
D: Diet
PA: Physical activity
HB: Human body and heart
E: Emotions management
IRT: Item Response Theory
