# Supplementary material for: Development and validation of a questionnaire to evaluate lifestyle-related behaviors in elementary school children
Source: BMC Public Health. 2015 Sep 16;15:901. doi: 10.1186/s12889-015-2248-6 (PMC4571115; doi:10.1186/s12889-015-2248-6)
Supplement: Additional file 1: — Appendix. (DOC 131 kb) [file 12889_2015_2248_MOESM1_ESM.doc]

Appendix

| **SI! PROGRAM FOR ELEMENTARY EDUCATION -.Data collection sheet.-** | |
| --- | --- |
| This is Alex, he was eating before leaving home… | |
| **Do you eat breakfast every day?** | **never**  **sometimes**  **always** |
| The school is very near to his house… | |
| **How is the healthiest way to go to school?** | **car**  **walking**  **underground/bus** |
| At school, the teacher was talking about the human body… | |
| **How much interest do you have in the human body and heart?** | **nothing**  **something**  **a lot** |
| **Choose an activity that can help your heart work better:** | **reading**  **watching TV**  **sport** |
| Then, they had to work on a project for biology class, but his classmate was having difficulty... | |
| **What do you do when your classmates have difficulty?** | ask the teacher for help  nothing  try to help him |
| **Should we cooperate in a school project?** | **never**  **sometimes**  **always** |
| Later, the bell rings for recess | |
| **Do you eat a mid-morning snack during recess?** | **never**  **sometimes**  **always** |
| **Do you play physically active games during recess?** | **never**  **sometimes**  **always** |
| Alex sometimes treats his classmates very harshly… | |
| **Do you treat your classmates harshly?** | **never**  **sometimes**  **always** |
| And sometimes, Alex’s classmates don't want him to play with them | |
| **How does he feel?** | **scared**  **sad**  **happy** |
| Today, they are playing tag | |
| **How does his heart beat after running?** | **slow**  **fast**  **always in an regular pattern** |
| At the end of the recess he is sweating | |
| **What is sweat made of?** | **blood**  **the** **fluids we drink**  **water and mineral salts** |
| After recess, the teacher always introduces some relaxation exercises | |
| **Do you ever practise any relaxation exercises?** | **never**  **sometimes**  **always** |
| During the physical education class, Álex makes a mistake passing the ball and a kid gets upset... | |
| **How do you feel when your friend is upset?** | **mad**  **remain indifferent**  **nervous** |
| Alex feels sad... | |
| **What do you do when you are sad?** | **be alone**  **tell to somebody**  **think about other things** |
| Lunchtime! | |
| **Do you eat lunch at school?** | **never**  **sometimes**  **always** |
| **Do you wash your hands before you eat?** | **never**  **sometimes**  **always** |
| Today his schoolmates try a new food (carrots) | |
| **Do you refuse to eat a new food you had never tried before?** | **never**  **sometimes**  **always** |
| **How often should you eat vegetables?** | **never**  **sometimes**  **every day** |
| **Do you like vegetables?** | **yes**  **no, but I eat them**  **no, and I refuse to eat them** |
| The main course is fish, | |
| **Which of these is a fish?** | **pear**  **steak**  **hake** |
| What's for dessert? Fruit! | |
| **Do you eat fruit or natural juices for dessert?** | **never**  **sometimes**  **always** |
| **How many servings of fruit do you eat each day?** | **I never eat fruit or drink any juice**  **1**  **≥ 2** |
| **Do you like fruit?** | **yes**  **no, but I eat them**  **no, and I refuse to eat them** |
| During the recess after lunch some children exercise | |
| **Which of these is an exercise?** | **move your body**  **read**  **play video games** |
| **Do you like to exercise?** | **never**  **sometimes**  **always** |
| **How does exercise affect your heart?** | **it’s good**  **it’s bad**  **it does not affect** |
| During the recess someone is teasing his friend | |
| **How do you react when someone teases your friend?** | **do nothing**  **try to help**  **fight** |
| Today it is someone’s birthday at school and candy treats are passed out at the end of the day | |
| **Do you wish you could have candy every day?** | **yes**  **just once a week**  **just for birthdays or parties** |
| **What do you usually do after school?** | **seating time**  **physical active** |
| After school Alex plays basketball | |
| **What do you usually do after school?** | **seating time**  **physical active** |
| **How do you feel when you have to exercise?** | **always happy**  **sometimes lazy**  **always lazy** |
| After school Alex plays basketball, and the defender passed the ball to him but he fails. The boy got upset and insults him. | |
| **Do you get upset when a friend fails?** | **never**  **sometimes**  **always** |
| **How do you react when someone insults you?** | **give insult back**  **report to an adult**  **talk with him/her** |
| After training they all drink some water | |
| **When should you drink every day?** | **if I’m thirsty**  **soda is better**  **when someone reminds me** |
| They were making jokes and a boy was blushing | |
| **Why do people blush?** | **embarrassment**  **happiness**  **sadness** |
| Some parents smoking at the school gate | |
| **What do you think about smoking?** | **don’t care**  **don´t like (say nothing** **ask not to smoke****)** |
| He always gets a sandwich on their way to home, but he prefers a muffin | |
| **Do you have a snack after school?** | **never**  **sometimes**  **always** |
| **When do you eat muffins or pastries?** | **every day**  **once a week**  **just for birthdays or parties** |
| Alex vacillated between staying at home or skating | |
| **You should rest when you are…** | **tired**  **bored**  **is not necessary** |
| Finally, he stayed at home and would to play videogames, but his parents do not allow him and they all played a board game (and he loses) | |
| **When your parents don't let you do what you want you...** | **get angry**  **try to understand why**  **obey directly** |
| **What is the best way to react when you lose?** | **just enjoy the game**  **understand why I lost**  **get upset** |
| Bath time! | |
| **What do you do when you have to shower or bath?** | **go directly**  **complain but go**  **get angry** |
| **How often do you take a shower or a bath?** | **every night**  **every other day**  **on weekends** |
| After the bath, Alex’s mother asked him to set the table and they dinner | |
| **What do you do when your parents ask you to set the table?** | **obey directly**  **complain but do**  **get angry and refuse** |
| **What should you eat daily to be healthy?** | **vegetables**  **legumes**  **muffins** |
| **Do you have dinner every day?** | **never**  **sometimes**  **always** |
| Bedtime! | |
| **When you have to go to bed you...** | **obey directly**  **complain but go**  **get angry and refuse** |
| **What can you do to get good sleep?** | **a story or a teddy bear**  **play video games**  **watch TV** |
| **Do you brush your teeth before bed?** | **always**  **when someone reminds me**  **never** |
| The weekend starts tomorrow! | |
| **What do you like doing on weekends?** | **stay at home**  **go to the park and play**  **go to the mall** |
| **What did you do last weekend?** *(****frequency physical active)*** | **rarely**  **sometimes**  **almost every weekend** |
| ***Posture check*** | **good posture**  **bad posture** |
